# Pharmacokinetics and Pharmacodynamics of Gamithromycin Treatment of *Pasteurella multocida* in a Murine Lung Infection Model

**DOI:** 10.3389/fphar.2019.01090

**Published:** 2019-09-24

**Authors:** Qingwen Yang, Xuesong Liu, Chenghuan Zhang, Kang Yong, Alancia Carol Clifton, Huanzhong Ding, Yun Liu

**Affiliations:** ^1^Heilongjiang Key Laboratory for Laboratory Animals and Comparative Medicine, Department of Veterinary Surgery, College of Veterinary Medicine, Northeast Agricultural University, Harbin, China; ^2^Laboratory of Veterinary Pharmacology, Department of Animal Science and Technology, Chongqing Three Gorges Vocational College, Chongqing, China; ^3^Laboratory of Veterinary Pharmacology, College of Veterinary Medicine, South China Agricultural University, Guangzhou, China

**Keywords:** gamithromycin, lung infection model, *Pasteurella multocida*, mice, pharmacokinetic/pharmacodynamic

## Abstract

Gamithromycin is approved for the treatment and prevention of bovine respiratory disease (BRD), which is caused mainly by *Mannheimia haemolytica, Pasteurella multocida, Histophilus somni*, and *Mycoplasma* species. In this study, multiple dosage regimens were administered to the neutropenic mouse lung infection model in order to investigate the pharmacokinetic/pharmacodynamic (PK/PD) parameters of gamithromycin treatment of *P. multocida* and to further define the PK/PD parameter that best correlates with the efficacy of gamithromycin against *P. multocida*. The PK characteristics of gamithromycin were analyzed after a single subcutaneous (s.c.) injection (1, 3, 6, and 9 mg/kg). The concentration–time profiles of unbound (*f*) gamithromycin in plasma samples were analyzed by non-compartmental analysis. The main PK parameters of gamithromycin for the area under the concentration–time curve from 0 to 24 h (*f* AUC_0–24_) and the peak drug concentration (*f C*_max_) values ranged from 0.86 to 8.42 µg·h/ml and from 0.55 to 5.69 µg/ml, respectively. The PD values were calculated based on multiple s.c. injections over 24 h (1, 3, 6, and 9 mg/kg at 6, 8, 12, and 24 h, respectively; total dosage 1–36 mg/ kg). The minimum inhibitory concentration (MIC) of gamithromycin against *P. multocida* in mice serum was 0.15 μg/ml. Analysis of PK/PD indices using the inhibitory effect *E*_max_ model indicated a strong correlation (*R* ^2^ = 0.9624) between the *f* AUC_0–24_/MIC ratio and various antibacterial effects. The area under the unbound concentration–time curve over 24 h to MIC (*f* AUC_0–24_/MIC) predicted for bacteriostatic action, 1-log_10_ reduction, 2-log_10_ reduction, and 3-log_10_ reduction were 56.77, 90.18, 143.06, and 239.44 h, respectively. These *in vivo* data may facilitate gamithromycin dosage optimization against *P. multocida* in veterinary medicine.

## Introduction

Bovine respiratory disease (BRD), which is one of the most common respiratory diseases in calves, occurs with high mortality and morbidity rates, leading to enormous economic losses in the cattle industry. The risk factors involved in BRD are diverse and complex, including mixing, transportation, viral and bacterial agents, natural immune responses, and others (Snowder et al., 2006; Dabo et al., 2007; Jones and Chowdhury, 2007; Snowder et al., 2007; Griffin et al., 2010). Although the pathogenesis of BRD is multifactorial, pathogenic bacteria, such as *Mannheimia haemolytica*, *Pasteurella multocida*, *Histophilus somni*, and *Mycoplasma* species, are the main contributing factors which contribute to morbidity and mortality (Portis et al., 2012).

Gamithromycin, a novel semi-synthetic macrolide, is approved for the treatment and prevention of BRD (Muraro et al., 2012; O’Connor et al., 2016). Like other macrolides, it plays a bacteriostatic and bactericidal role by inhibiting the ribosomal 50S subunit. Prior studies have demonstrated that gammamycin has high antibacterial activity against *Mycoplasma mycoides*, *M. haemolytica*, and *P. multocida* (Rossi et al., 2010; Baggott et al., 2011; Forbes et al., 2011; Mitchell et al., 2013; Abell et al., 2017; Snyder et al., 2017). Pharmacokinetic (PK) data for gamithromycin delivered by subcutaneous (s.c.) injection have been reported in different species, such as foals, sheep, cattle, broiler chickens, and pigs (Watteyn et al., 2013; Kellermann et al., 2014; Wyns et al., 2014; Jones et al., 2015; Berlin et al., 2017).

Selecting effective PK/pharmacodynamic (PD) indices for analysis is crucial for optimization of the dose regimens in veterinary medicine (Toutain et al., 2002). PK/PD of gamithromycin has been investigated in cattle and turkey poults (Dedonder et al., 2015; Watteyn et al., 2015). Previous study has demonstrated that higher PK/PD indices showed the strongest correlation to positive treatment outcomes (Dedonder et al., 2015). However, there are no reports of the *in vivo* PK data combined with *in vivo* PD for the evaluation of the antibacterial activity of gamithromycin against *P. multocida* using a neutropenic murine lung infection model, which is commonly used to investigate the relationship between host, drug, and pathogens (Andes and Craig, 2006; Ferran et al., 2011; Qu et al., 2015; So et al., 2015; Thabit et al., 2016; Zhou et al., 2017b; Zeng et al., 2018). This relatively stable and mature model avoids the impact of the host immune system on antimicrobial efficacy. Using this model for analysis of the *in vivo* changes in unbound drug concentration in plasma indicated that changes in the amount of bacteria were highly related to the antibacterial activity of the drug. For macrolides, the neutropenic mouse lung infection model was used to evaluate the antibacterial activity of tildipirosin against *P. multocida* (Zeng et al., 2018).

In this study, we investigated the PK and PD characteristics of gamithromycin against *P. multocida* in a neutropenic murine lung infection model and determined the value of PK/PD index to achieve various antibacterial activity.

## Materials and Methods

### Antibiotics and Bacteria

Gamithromycin standard (purity 98.6%) and d5-gamithromycin (purity: 93.7%) was provided by Zhong sheng tiao zhan Biotechnology, Co., Ltd (Tianjin, China). Cyclophosphamide was purchased from Aladdin, Co., Ltd (Shanghai, China). ICR mice plasma was purchased from Ruite Biotechnology, Co., Ltd (Guangzhou, China). *P. multocida* NM-5-7, isolated from a yellow cattle which died of hemorrhagic septicemia in Neimenggu province, was identified by matrix-assisted laser desorption/ionization time-of-flight (MALDI-TOF)/mass spectrometry (MS) analysis (AXIMA Assurance, Shimadzu). Six clinical strains of *P. multocida* (NU01–NU06) were provided by the Department of Preventive Veterinary Medicine, College of Veterinary Medicine, Northeast Agricultural University.

### Animals

Specific-pathogen-free (SPF) female ICR mice (aged 6 weeks; 33–37 g) were purchased from Guangdong Medical Lab Animal Center (Guangzhou, China). Mice were maintained with SPF food and water for 1 week.

### *In Vitro* Susceptibility Studies

The minimum inhibitory concentration (MIC) of gamithromycin against P. multocida was determined in serum and Mueller–Hinton broth (MHB) using Clinical and Laboratory Standards Institute microdilution methods. Briefly, a minimum of 10 freshly cultured colonies were transferred into MHB and incubated at 37°C on a shaking incubator (220 rpm) for 5 h [approximately 8 log colony-forming unit (CFU)/ml]. Culture aliquots of 100 µl were added into a 96-well plate to obtain a series of twofold-dilution drug concentrations. Three overlapping sets of doubling dilutions were used to improve the accuracy of the MIC determinations (0.025–0.8, 0.031–1, and 0.038–1.2 µg/ml). The MIC was defined as the lowest concentration of gamithromycin that inhibited the visible bacterial growth in serum and MHB. Susceptibility testing was performed in triplicate, and *Staphylococcus aureus* ATCC 29213 was used as control strain.

### Neutropenic Mouse Lung Infection Model

The neutropenic mouse lung infection model was established as previously described (Ferran et al., 2011; Qu et al., 2015; Zeng et al., 2018). Briefly, neutropenic mice were successfully produced (neutrophil count < 100 mm^3^) after intraperitoneal cyclophosphamide injection (150 mg/kg daily for 4 days followed by one dose at 100 mg/kg on day 5). Lung infection was achieved by tracheal administration of *P. multocida* NM-5-7. Following anesthesia with pentobarbital sodium, an intravenous catheter (22 G, “Y” type, without mandrel) was inserted into the trachea of neutropenic mice, and 50 µl of bacterial suspension (approximately 8 log_10_ CFU/ml) was delivered. The mice were then placed in an inverted position for approximately 15 s. The neutropenic mouse lung infection model was established, when the bacterial burden reached 6 log_10_ CFU per lung.

### PK Analysis of Gamithromycin in Neutropenic Lung Infected Mice

Approximately 3 h after bacterial inoculation, when the bacterial burden was 6.12 ± 0.08 log_10_ CFU per lung, 200 µl of gamithromycin was administered using single s.c. doses of 1, 3, 6, and 9 mg/kg. Sedation management was administered as previously described (Zeng et al., 2018). Briefly, the mice were placed in an induction chamber with an oxygen flow rate of 0.5–1.0 L/min. Isoflurane vapor (3–5%) was applied for induction and then reduced (1–3%) for maintenance. Retro-orbital blood samples were collected into 1.5-ml plastic tubes (containing heparin sodium) at 0.083, 0.167, 0.25, 0.5, 0.75, 1, 2, 3, 6, 9, 12, and 24 h after treatment (*n* = 5). Each mouse was sampled at three or four time points. This was decided based on a trade-off between reducing inter-animal variability to obtain accurate PK data, reducing the number of mice used and to adhere to acceptable welfare conditions. Plasma was isolated following centrifugation (4,500 × *g* for 10 min at 4°C) and stored at −80°C for analysis.

Plasma concentrations of gamithromycin were analyzed as previously described (Dedonder et al., 2015). Briefly, 100 µl of each plasma sample was added to 10 µl of d5-gamithromycin (1 µg/ml) and 400 µl of formic acid (1% in acetonitrile) and then vortexed for 1 min. After centrifugation (3,500 × *g* for 10 min at 4°C), 200 µl of supernatant was diluted with 600 µl of ultrapure water for liquid chromatography–tandem mass spectrometry (LC-MS/MS) analysis of gamithromycin concentrations by extrapolation against a standard calibration curve (0.01–4 μg/ml). The limit of detection (LOD) and limit of quantification (LOQ) values for this assay were 0.005 and 0.01 µg/ml, respectively. The recoveries of gamithromycin in plasma samples were >85%. All inter- and intra-assay variations measured by calculation of relative standard deviation (%RSD) were <10%.

Plasma PK parameters of gamithromycin, such as the elimination half-life (t_1/2_), the time of maximum concentration (*T*_max_), the area under the concentration–time curve from 0 to 24 h (*f* AUC_0–24_), and the peak drug concentration (*f* *C*_max_), were analyzed by non-compartmental analysis (WinNonlin 5.2.1, Pharsight Corporation, Mountain View, CA, USA). PK parameters were represented as means *±* standard deviation (SD).

### *In Vitro* Binding of Gamithromycin to Plasma Proteins

The protein binding in mice plasma was determined by ultrafiltration methods as previously described (Huang et al., 2010). Briefly, plasma was spiked with appropriate amounts of gamithromycin to achieve final concentrations of 0.01, 0.4, 1, and 4 µg/ml and then vortexed and incubated at 35 ± 2°C for 30 min. Aliquots of 1 ml of the plasma mixtures were then transferred to Millipore Centrifree ultrafiltration devices and centrifuged at approximately 1,730 × *g* for 30 min at 20°C. Subsequently, 10 µl of d5-gamithromycin (1 µg/ml) was added to 100 µl of each filtrate and determined using the above-mentioned method. Plasma mixtures of 100 µl without ultrafiltration procedures were also determined using the same method. Three separate sets of experiments were undertaken for each concentration. The drug percentage bounded to protein was calculated as follows:

$$\%\;\mathrm{Bound}\;=\left(\frac{\mathrm{concentration in plasma}-\mathrm{concentration in filtrate}}{\mathrm{concentration in plasma}}\right)\times 100$$

### PD Study in the Neutropenic Mouse Lung Infection Model

Following the successful establishment of the neutropenic lung infection model, mice were divided randomly into 16 experimental groups (*n* = 3 per group). The experimental groups were treated with gamithromycin *via* s.c. injection 3 h after P. multocida NM-5-7 inoculation; this was designated as the 0-h time point for treatment. The dosing regimens were 1, 3, 6, and 9 mg/kg every 6, 8, 12, and 24 h, respectively. The range of gamithromycin for this study was from 1 to 36 mg/kg per 24 h. All mice in experimental groups were euthanized by CO_2_ asphyxiation 24 h after treatment. Mice in the untreated control group were sacrificed prior to the 0-h time point for gamithromycin treatment and 24 h after treatment (*n* = 3 per time point). The CFU in each homogenized lung sample was determined after sacrifice as previously described (Qu et al., 2015). Briefly, all lung samples were aseptically removed and homogenized in sterilized saline (5 ml). The homogenates were then diluted (10-fold serial dilutions). Twenty microliters of each lung homogenate dilution was dropped onto 5% sterile defibrinated sheep blood trypticase soy agar (5% BTSA). The homogenate dilution was dropped in triplicate. Finally, the bacterial colonies were counted on the 5% BTSAs after incubation for 18–24 h at 37°C, and the mean values were used for data analysis. The LOD was 50 CFU/ml.

### PK/PD Integration and Modeling

The PK parameters of gamithromycin were calculated using WinNonlin software version 5.2.1 (Pharsight Corporation), after single s.c. injections of 1, 3, 6, and 9 mg/kg. The non-compartmental analysis was performed, and the extravascular input best reflected the unbound plasma concentration–time profiles of gamithromycin. The PK/PD indices were calculated using the inhibitory effect *I*_max_ model according to the following equation:

$$E=E_{0}-\frac{I_{\max}\times X}{{\mathrm{IC}}_{50}+X}$$

where *E* represents the antibacterial effect, defined as the change between the treated mice after 24 h and the initial untreated control mice; *I*_max_ denotes the maximum change in log_10_ CFU per lung after 24-h treatment with gamithromycin; *E*_0_ indicates the change in log_10_ CFU per lung in control samples between time 0 and 24 h; *IC*_50_ represents the *X* value producing 50% of the *I*_max_, where *X* indicates the PK/PD index (e.g. *f* AUC_0–24_/MIC, *f C*_max_/MIC, and %T > MIC). PD indices were analyzed by non-linear regression using WinNonlin software. Due to regression in each of the PK/PD indices, in addition to *R* ^2^, the percentage of the variance explained by the model is given by *R* ^2^.

### Statistical Analyses

Statistical analyses were done by using analysis of variance, and significant differences were analyzed using Bonferroni’s correction for inter-group comparisons. Differences were considered significant when *P* < 0.05.

## Results

### *In Vitro* Susceptibility Testing

The MICs of gamithromycin against *P. multocida* in MHB and serum are shown in **Table 1**. The average MICs in MHB, mouse serum, and adult bovine serum were 0.71, 0.107, and 0.036 µg/ml, respectively. The average MIC in mouse serum was threefold higher than that in adult bovine serum.

**Table 1:** The minimum inhibitory concentration (MIC) of gamithromycin against *Pasteurella multocida* in Mueller–Hinton broth (MHB) and serum.

*P. multocida* strain	MHB (µg/ml)	Bovine serum (µg/ml)	Mouse serum (µg/ml)	MHB/serum MIC ratio		
				Bovine	Mouse	Mouse/bovine
*P. multocida* NM-5-7	0.5	0.0313	0.15	16	3	5
NU01	1	0.0313	0.075	32	13	2
NU02	0.5	0.0313	0.075	16	7	2
NU03	1	0.0625	0.15	16	7	2
NU04	0.5	0.0313	0.075	16	7	2
NU05	0.5	0.0313	0.15	16	3	5
NU06	1	0.0313	0.075	32	13	2
**Average**	0.71	0.036	0.107	20	8	3

### *In Vitro* Binding of Gamithromycin to Plasma Proteins

The protein binding of gamithromycin in mouse plasma with a concentration range of 0.01–4 µg/ml is shown in **Table 2**. The average binding was found to be 27.2 ± 1.8%.

**Table 2:** Percentage of bound gamithromycin in plasma of mice.

Concentration (µg/ml)	Lot 1	Lot 2	Lot 3	Average^(A)^
0.01	26.5	29.8	27.2	27.8 ± 1.4
0.04	23.8	22.5	29.5	25.3 ± 3.0
1	25.8	27.9	23.6	25.8 ± 1.8
4	31.2	30.3	27.7	29.7 ± 1.5
**Average^(B)^**	26.8 ± 2.7	27.6 ± 3.1	27.0 ± 2.1	27.2 ± 1.8^(C)^

^(A)^Numbers are the average ± standard deviation of three lots of plasma.

^(B)^Numbers are the average ± standard deviation of four concentrations.

^(C)^Value is the average ± standard deviation of the four concentrations from the average of the three lots.

### PK of Gamithromycin in Neutropenic Lung Infected Mice

**Figure 1:**
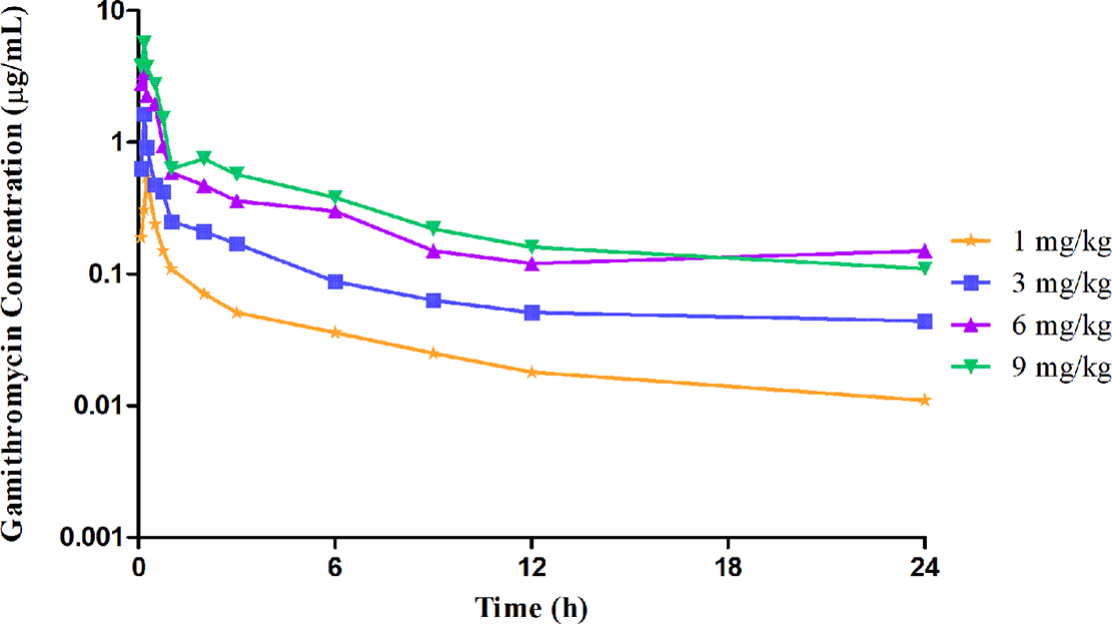
Unbound plasma gamithromycin concentration–time courses in neutropenic infected mice following single subcutaneous injections of 1, 3, 6, and 9 mg/kg.

The unbound plasma gamithromycin concentration–time profiles in neutropenic lung infected mice after single s.c. injection at 1, 3, 6, and 9 mg/kg are presented in **Figure 1**. Unbound plasma gamithromycin concentrations were calculated based on a plasma protein binding activity of 27.2%. The main PK parameters are shown in **Table 3**. The *f* AUC_0–24_ and *f* *C*_max_ values ranged from 0.86 to 8.42 µg·h/ml and 0.55 to 5.69 µg/ml, respectively. Both the *f* AUC_0–24_ and *f C*_max_ values increased with dose escalation (1–9 mg/kg). Moreover, the t_1/2_ values ranged from 8.04 to 13.64 h.

**Table 3:** The pharmacokinetic parameters of gamithromycin in neutropenic lung infected mice plasma after single subcutaneous (s.c.) injection dose levels at 1, 3, 6, and 9 mg/kg of body weight.

Parameters (units)	Dose levels			
	1 mg/kg	3 mg/kg	6 mg/kg	9 mg/kg
*f* t_1/2_ (h)	13.64	9.07	9.43	8.04
*T*_max_ (h)	0.25	0.17	0.17	0.17
*f* *C*_max_ (µg/ml)	0.55	1.65	3.4	5.69
*f* AUC_0–24_ (µg·h/ml)	0.86	2.37	6.33	8.42

f C_max_, maximum unbound gamithromycin concentration in plasma; T_max_, time to achieve maximum unbound gamithromycin concentration in plasma; f t_1/2_, elimination half-life; f AUC_0−24_, area under the unbound plasma gamithromycin concentration–time curve over 24 h.

### PK/PD Integration and Modeling

**Figure 2:**
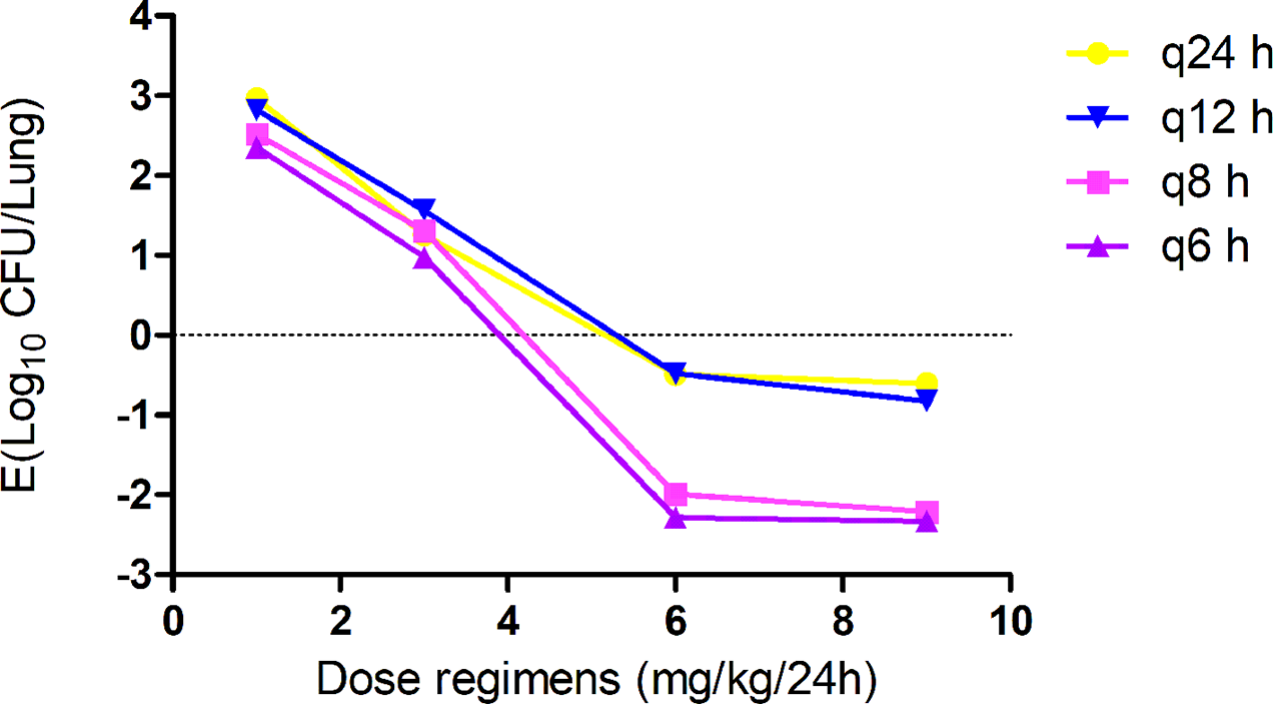
*In vivo* dose fractionation with gamithromycin using the murine neutropenic lung infection model. Each symbol represents the mean value of lung bacterial infection from three mice infected with *Pasteurella multocida* NM-5-7. Sixteen dose regimens of gamithromycin were used to treat the *P. multocida* NM-5-7 infection. The change in the log_10_ number of colony-forming unit (CFU)/lung was measured at the start and after 24 h of therapy. Data points below the horizontal dashed line represent killing, and points above the horizontal dashed line represent growth.

**Figure 3:**
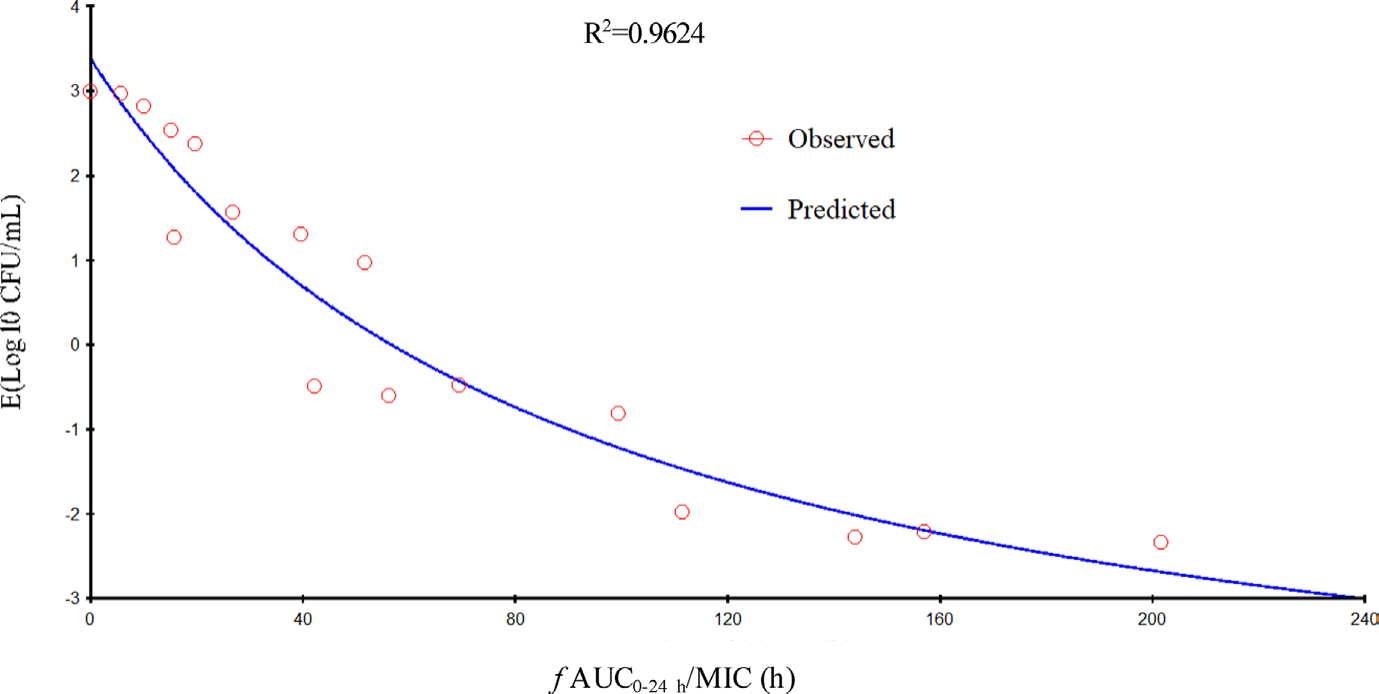
Relationship of *f* AUC_24_/minimum inhibitory concentration (MIC) for *Pasteurella multocida* NM-5-7 with the change in the log_10_ number of colony-forming unit (CFU)/lung after 24 h of therapy. *R* ^2^ is the correlation coefficient.

**Figure 4:**
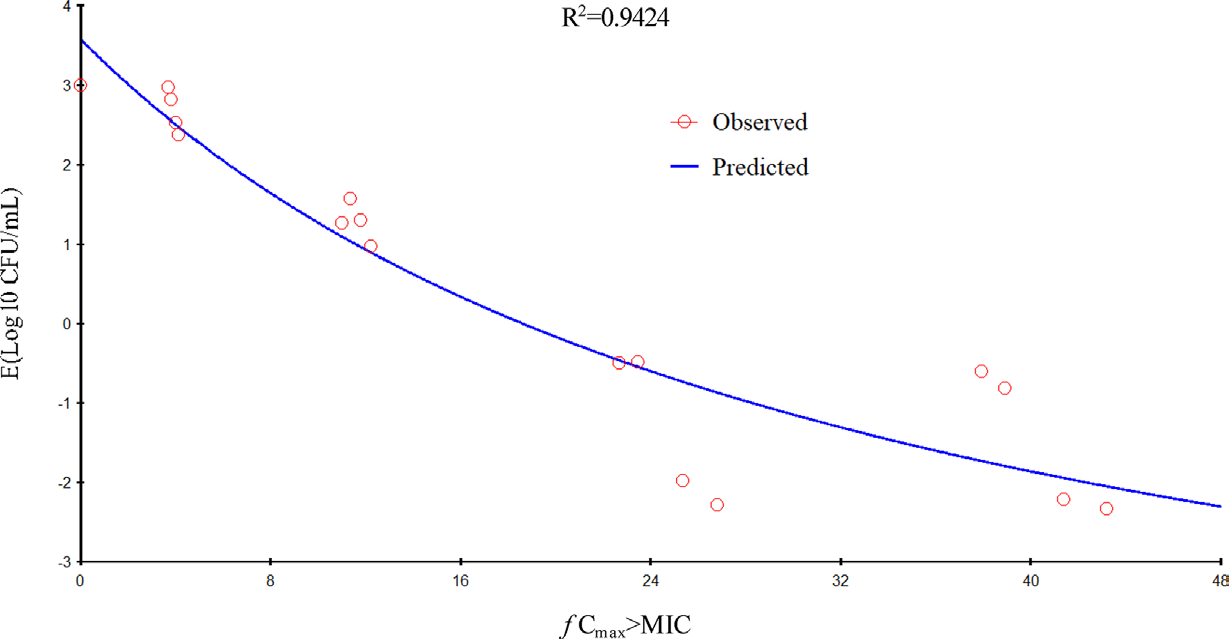
Relationship of *f* C_max_/minimum inhibitory concentration (MIC) for *Pasteurella multocida* NM-5-7 with the change in the log_10_ number of colony-forming unit (CFU)/lung after 24 h of therapy. *R* ^2^ is the correlation coefficient.

Gamithromycin was administered when the bacterial load reached 6.12 ± 0.08 log_10_ CFU/lung approximately 3 h after inoculation. In the untreated control group, the bacterial load reached 8.91 ± 0.03 log_10_ CFU per lung across the 24-h treatment period. The maximum antibacterial effect in experimental groups was reduced to a rate of 2.33 ± 0.05 log_10_ CFU per lung 24 h after treatment. The fractionated dose regimen curves were consistent, indicating that the *f* AUC_0–24_/MIC ratio is the predictive PD index (**Figure 2**). The main PK/PD indices are shown in **Table 4**. In this model, the *f* AUC_0–24_/MIC ratio was regarded as the best PK/PD index of the antibacterial effect (*R* ^2^ = 0.9624). Correlations between PK/PD indices and antibacterial effects are presented in **Figures 3** and **4**.

**Table 4:** The pharmacokinetic/pharmacodynamic (PK/PD) parameter estimates for the *f* AUC_0–2__4_/MIC to achieve various antibacterial effects.

Parameters (units)	Values
*E*_0_ (log_10_ CFU/lung)	3.38
*I*_max_ (log_10_ CFU/lung)	8.81
*IC*_50_ (h)	91.25
*f* AUC_0–24_/MIC for bacteriostatic (h)	56.77
*f* AUC_0–24_/MIC for 1-log_10_ reduction (h)	90.18
*f* AUC_0–24_/MIC for 2-log_10_ reduction (h)	143.06
*f* AUC_0–24_/MIC for 3-log_10_ reduction (h)	239.44

I_max_ denotes the maximum change in log_10_ CFU/lung after 24-h treatment with gamithromycin; E_0_ indicates the change in log_10_ CFU/lung in control samples between time 0 and 24 h; IC_50_ represents the f AUC_0–24 h_/MIC value producing 50% of the I_max_. AUC, area under the curve; CFU, colony-forming units; MIC, minimum inhibitory concentration.

## Discussion

PK studies of gamithromycin in many different animals, including foals, cattle, broiler chickens, sheep, and pigs indicate that gamithromycin shares characteristics with macrolides, including rapid absorption, wide distribution, and prolonged half-life (Watteyn et al., 2013; Kellermann et al., 2014; Wyns et al., 2014; Jones et al., 2015; Berlin et al., 2017). In this present study, the *T*_max_ of plasma gamithromycin was 0.17 h in mice after a single s.c. injection at a recommended dosage of 6 mg/kg, which was higher than values obtained using the same dosage regimen in cattle (1 h), sheep (0.911 h), and pigs (0.63 h) (Huang et al., 2010; Kellermann et al., 2014; Wyns et al., 2014). Slightly higher values were obtained in broiler chickens (0.13 h) (Watteyn et al., 2013). Inter-species differences might be the major reason for these different outcomes. In the current study, both the *f* AUC_0–24_ and *f C*_max_ values were proportional to the dose, similar results were also previously reported in neutropenic lung infected mice model for tildipirosin against *P. multocida* (Zeng et al., 2018). Moreover, the *f* AUC_0–24_ and *f* *C*_max_ values 6.33 µg·h/ml and 3.4 µg/ml respectively, were higher than those obtained in tildipirosin against *P. multocida*, after using the same dosage at 6 mg/kg.

The combination of PK and PD used in predicting the antimicrobial activity of antimicrobial agents and calculating the dosage of antibiotics to achieve different antimicrobial efficacy is regarded as a very useful method to optimize the dosage regimen in veterinary clinical settings (Toutain et al., 2002; Lees et al., 2006). Therefore, it is very important to select effective PK/PD indices for optimization of the dose regimens. Macrolides are usually classified as time-dependent antimicrobial agents. In a previous study, the PK/PD index %*T* > MIC showed the strongest correlation with antibacterial activity (Ahmad et al., 2016). However, for the second generation of macrolides, including tulathromycin and tildipirosin, the *f* AUC_0–24_/MIC ratio has been reported as the best PK/PD index used to describe the antibacterial effect against *P. multocida* (Zhou et al., 2017a; Zeng et al., 2018). A previous study investigated tulathromycin against *P. multocida* in a porcine tissue cage model; results indicated that AUC_0–24_/MIC exhibited a high correlation with the antibacterial effect in serum and the values of AUC_0–24_/MIC for bacteriostatic, 3-log_10_ reduction, and 4-log_10_ reduction were 44.55, 73.19, and 92.44 h, respectively (Zhou et al., 2017a). Another study explored tildipirosin against *P. multocida* in a neutropenic lung infected mice model, suggesting that the respective values for bacteriostatic action, 1-log_10_ reduction, and 2-log_10_ reduction were achieved when *f* AUC_0−24_/MIC reached 19.93, 31.89, and 53.27 h, respectively (Zeng et al., 2018). These previous studies highlighted that the second generation of macrolides had a strong antimicrobial activity against *P. multocida*. However, for one of the second generation of macrolides, the efficacy of gamithromycin against *P. multocida* has not been studied. Therefore, a neutropenic lung infected mice model was used to evaluate the efficacy of gamithromycin against *P. multocida*.

In the current study, gamithromycin dose fractionation experiments during the 24-h treatment period showed that the correlation indices (*R* ^2^ values) of the *f* AUC_0–24_/MIC, the *f C*_max_/MIC, and the %*T* > MIC were 0.9624, 0.9424, and 0.8725, respectively. Based on the *R* ^2^ values, the *f* AUC_0–24_/MIC ratio was more predictive of the antimicrobial effect, which was similar to previous studies. The current study suggested that the *f* AUC_0−24_/MIC ratios required for bacteriostatic action, 1-log_10_ reduction, and 2-log_10_ reduction were 56.77, 90.18, and 143.06 h, respectively, which were higher than estimated in a prior study (Zeng et al., 2018). Different drug and different strains of MIC might contribute to these variances.

Previous studies have shown that MICs in MHB differ from those in biological sample matrices, indicating that MICs in MHB were artificially high (Zhou et al., 2017a; Zeng et al., 2018). Thus, it is important to use the MIC obtained in biological sample matrices rather than in MHB, as this may be considered more suitable for macrolide PK/PD integration (Lees et al., 2006; Toutain et al., 2017). To investigate the effect of serum on the determination of MICs, we determined the MICs of P. multocida NM-5-7 and six other *P. multocida* strains in serum and in MHB. The results showed that MICs of *P. multocida* were 26-fold lower in adult bovine serum than in MHB, when we take the protein binding ratio of 26% into account. Compared with other strains and macrolides, the MIC ratio of gamithromycin against P. multocida was much lower than that of gamithromycin against *M. mycoides* (Mitchell et al., 2013) and that of tulathromycin against P. multocida (Toutain et al., 2017). The susceptibility of different strains to different drugs leads to these results.

In the area of human medicine, the neutropenic mouse model is commonly used to predict the therapeutic dose for macrolides (Tessier et al., 2002; Hoffman et al., 2003). Thus, in the current study, we could extend to predict a daily dosage in cattle using the following equation: Dose = [Cl × (AUC_0–24_/MIC) × MIC_90_]/(*F* × fu) (Toutain et al., 2002). Combining the current data and previous data on cattle reported by Huang et al. (Cl = 712 ml/kg/h, fu = 0.74, and *F* = 1), daily dosages to achieve bacteriostatic effect, 1-log_10_ reduction, 2-log_10_ reduction, and 3-log_10_ reduction were 2.10, 3.34, 5.29, and 8.86 mg/kg, respectively. When we consider the effect of serum, more realistic doses were predicted for currently observed MICs in cattle, indicating that the MIC obtained in bovine serum was more suitable for macrolide PK/PD integration for cattle. Some evidence could be found in previous studies, which confirmed that the presence of natural media, such as serum and plasma, could reduce the MIC of bacteria by altering the integrity of gram-negative bacterial outer membrane and increasing outer-membrane permeability, thus increasing the accumulation of macrolides (Buyck et al., 2012; Mustafa et al., 2017). Obviously, the daily dosage (8.86 mg/kg) required for 3-log_10_ reduction was much higher than the recommended dose (6 mg/kg), indicating that the recommended dose would fail to achieve an eradication effect. However, for long-acting injection, a dose required for 2-log_10_ reduction could be enough to reduce the incidence of disease (Toutain et al., 2017). Therefore, the recommended dose of gamithromycin would be effective in the treatment of *P. multocida* infection in veterinary clinical medicine. But, it is still worth remembering that these theoretical values still need validation in clinical practice.

A few limitations of this model should be noted. First, the clinical characteristics of laboratory infections differ from those of the natural infection. In the present study, only *P. multocida* was used for modeling, ignoring the clinical influences of other possible bacterial pathogens (*M. haemolytica* and *H. somni*) in BRD. The second limitation is the existence of individual variation, which might have contributed to variation in the results. Third, the PK values obtained in plasma concentration–time profiles were used for PK/PD integration and modeling. However, these drug concentrations do not accurately reflect the actual real-time concentrations in the bacterial microenvironment. Further studies should take these factors into consideration.

## Conclusion

In the present study, a neutropenic mouse lung infection model was first used to evaluate the *in vivo* PK and PD indices of gamithromycin. The *f* AUC_0−24_/MIC ratio showed the strongest correlation with antibacterial activity. Furthermore, the current study suggests that the bacteriostatic action, 1-log_10_ reduction, 2-log_10_ reduction, and 3-log_10_ reduction of the bacterial count were achieved when the *f* AUC_0–24_/MIC ratio reached 56.77, 90.18, 143.06, and 239.44 h, respectively. Our research provides useful pharmacological data which could assist in optimizing the clinical use of gamithromycin against infections caused by *P. multocida*.

## Data Availability

The raw data supporting the conclusions of this manuscript will be made available by the authors, without undue reservation, to any qualified researcher.

## Ethics Statement

This study was carried out in accordance with the recommendations of the Committee on the Ethics of Animals of the South China Agricultural University and was performed according to the American Association for Accreditation of Laboratory Animal Care guidelines. The protocol was approved by the Committee on the Ethics of Animals of the South China Agricultural University (approval number: 2017047).

## Author Contributions

YL and HD conceived and designed the experiments. QY, XL, and KY performed the experiments. CZ analyzed the data. QY drafted the manuscript. AC contributed to the revision. All authors read and approved the final manuscript.

## Funding

This work was supported by the National Key Research and Development Program of China (2016YFD0501310).

## Conflict of Interest Statement

The authors declare that the research was conducted in the absence of any commercial or financial relationships that could be construed as a potential conflict of interest.
